# Molecular and hormonal mechanisms of male infertility and their clinical implications

**DOI:** 10.6026/973206300220089

**Published:** 2026-01-31

**Authors:** Humaira Alam, Fahmida Zabin, Kazi Farhana Begum, Nigar Sultana, Taqik A Ibrahim

**Affiliations:** 1 Department of Reproductive Endocrinology and Infertility, Bangladesh Medical University, Shahbag, Dhaka, Bangladesh; 2 Department of Obstetrics and Gynaecology, Bangladesh Medical University, Shahbag, Dhaka, Bangladesh; 3 Department of Molecular and Cellular Biology, University of Guelph, Guelph, Canada

**Keywords:** Male infertility, hormonal imbalance, oxidative stress, sperm DNA fragmentation, Bangladesh

## Abstract

Male infertility is a pressing health issue in Bangladesh, affecting a significant portion of the male population and necessitating investigation into its underlying molecular and hormonal mechanisms. Therefore, it is of interest to investigate the molecular and hormonal mechanisms underlying male infertility in Bangladesh, a pressing health issue affecting a significant portion of the male population. Hormonal assays and molecular biomarkers, including sperm DNA fragmentation, reactive oxygen species (ROS) and HSP70 expression, were analyzed. Infertile men showed show FSH and LH levels, reduced testosterone and elevated ROS and DNA fragmentation compared to controls. Thus, novel insights into the molecular and hormonal mechanisms contributing to male infertility in Bangladesh specifically highlight the impact of hormonal imbalance and oxidative stress.

## Background:

Male infertility is a major health problem, affecting approximately 7% of the global male population and contributing to nearly half of all infertility cases worldwide [1]. Infertility has wide-ranging psychosocial and economic consequences, particularly in developing countries such as Bangladesh, where cultural expectations around childbearing often lead to stigma for affected men. Despite significant progress in reproductive medicine, the underlying mechanisms of male infertility remain poorly understood in many low- and middle-income regions. The regulation of male fertility depends heavily on the hypothalamic-pituitary- gonadal (HPG) axis. Gonadotropins such as follicle-stimulating hormone (FSH) and luteinizing hormone (LH) are critical for spermatogenesis and testosterone biosynthesis. Dysregulation of this axis results in impaired sperm production and compromised fertility [2]. Late-onset hypogonadism is a recognized clinical entity in which testosterone production declines, often associated with increased gonadotropins, metabolic abnormalities and reduced sexual function [3]. Pharmacological interventions, such as aromatase inhibitors, have been studied as therapeutic options to correct testosterone-estradiol imbalance in infertile men, highlighting the significance of hormonal modulation [4]. Estrogens also play important roles in male reproductive physiology. Both genomic and non-genomic estrogen actions influence sperm function, capacitation and acrosome reactions [5]. Abnormal estradiol signaling, as observed in some infertile men, may impair spermatogenesis by exerting negative feedback on gonadotropin release. This interplay between testosterone and estradiol is critical for maintaining optimal sperm production. Beyond hormonal dysregulation, oxidative stress represents a major molecular mechanism of infertility. Reactive oxygen species (ROS), when produced in excess, damage sperm membranes, mitochondrial integrity and DNA are ultimately impairing fertilization potential [6]. Lifestyle factors, such as smoking, significantly exacerbate oxidative stress, with meta-analyses consistently linking tobacco exposure to reduced semen quality and motility [7]. Sperm DNA fragmentation is now widely recognized as a biomarker of impaired fertility, with clinical guidelines recommending its assessment in selected patients. Elevated DNA fragmentation has been associated with poor fertilization, abnormal embryonic development and recurrent pregnancy loss [8]. Heat shock proteins and other stress-response pathways are also involved in sperm quality regulation, reflecting the molecular stress burden on germ cells. Obesity and metabolic dysfunction further contribute to male infertility. Excess adiposity alters sex hormone levels through increased aromatization of androgens to estrogens, reduced testosterone and heightened oxidative stress [9]. Population-based studies, such as the LIFE study, have demonstrated strong associations between increased body mass index (BMI), waist circumference and reduced semen quality [10]. In Bangladesh, where obesity and diabetes are rising, these findings are particularly relevant. Environmental and occupational exposures, including heavy metals, have also been implicated in impaired male reproductive functions, underscoring the multifactorial nature of the problem [11]. Male infertility is often idiopathic, with no clear clinical cause identified despite extensive evaluation. Advances in molecular biology have revealed genetic and epigenetic alterations as possible contributors. Studies on idiopathic infertility highlight disruptions in sperm chromatin integrity, mitochondrial function and epigenetic regulation [12]. These molecular insights are critical to developing targeted diagnostic and therapeutic approaches. Therefore, it is of interest to evaluate the hormonal profiles and molecular biomarkers of infertile men in Bangladesh compared with fertile controls.

## Methodology:

This case-control study was conducted at the Department of Reproductive Endocrinology and Infertility, Bangladesh Medical University (BMU), Dhaka, Bangladesh and in several private hospitals in Dhaka, between January and December 2024. A total of 300 men were included: 200 diagnosed with infertility and 100 fertile controls.

## Sample selection:

## Inclusion criteria:

[1] Men aged 20-45 years.

[2] For cases: history of primary or secondary infertility of ≥1 year, confirmed by semen analysis.

[3] For controls: proven fertility (father of at least one child within the past 2 years).

## Exclusion criteria:

[1] History of varicocele surgery or orchitis.

[2] Chronic systemic illnesses (e.g., renal or hepatic failure).

[3] Current use of anabolic steroids, hormonal therapy, or chemotherapy.

[4] Refusal to provide informed consent.

## Data collection and study procedure:

Informed consent was obtained from all participants. Data were collected using structured questionnaires, clinical examinations and laboratory assessments. Venous blood samples were analyzed for FSH, LH, testosterone, prolactin and estradiol using ELISA kits. Semen samples were collected after 3-5 days of abstinence and assessed according to WHO criteria. Sperm DNA fragmentation was measured using the sperm chromatin dispersion test, while reactive oxygen species levels were quantified via chemiluminescence assay. Data were analyzed using SPSS version 25.0. Independent t-tests were applied for continuous variables and chi-square tests for categorical variables. Statistical significance was set at p ≤ 0.05. Confidentiality was strictly maintained.

## Results:

Table 1 presents the baseline characteristics of the study population. Infertile men had a higher mean body mass index (27.1±3.4 kg/m^2^) compared to fertile controls (24.6±2.9 kg/m^2^). The mean duration of infertility among cases was 3.2±1.8 years. Diabetes mellitus was more prevalent among infertile men (14%) than in controls (6%). Table 2 describes the hormonal profiles of the two groups. Infertile men demonstrated significantly elevated FSH (9.1±3.7 mIU/mL) and LH (6.4±2.3 mIU/mL) levels compared to controls. Serum testosterone levels were markedly reduced in the infertile group (3.1±1.2 ng/mL) relative to controls (5.2±1.6 ng/mL). Prolactin and estradiol levels were significantly higher in infertile men. Table 3 shows molecular biomarkers associated with infertility. Infertile men exhibited increased sperm DNA fragmentation (32.5±9.1%) compared to controls (15.6±6.4%). Reactive oxygen species (ROS) levels were nearly double in infertile men. Furthermore, mitochondrial membrane potential loss and HSP70 expression were both significantly higher in the infertile group. Table 4 summarizes the hormonal and molecular dysregulations. Among infertile men, 38% had low testosterone, 29% had elevated FSH and 44% had ROS levels exceeding normal thresholds. Over one-third (36%) demonstrated high DNA fragmentation, suggesting strong associations between hormonal a nd molecular alterations and impaired fertility potential.

## Discussion:

Male infertility is a complex disorder resulting from the interplay of hormonal dysregulation and molecular disturbances. In this study, infertile Bangladeshi men demonstrated significantly higher gonadotropin levels, reduced testosterone and elevated estradiol and prolactin, consistent with prior reports of endocrine disruption in male infertility [4]. Elevated FSH and LH levels suggest impaired testicular function, particularly Sertoli and Leydig cell dysfunction, which is a hallmark of primary testicular failure [2]. The observed reduction in testosterone aligns with findings from Huhtaniemi, who described late-onset hypogonadism as a contributor to subfertility [3]. Testosterone deficiency directly affects spermatogenesis, sperm motility and libido. Elevated estradiol levels further exacerbate this imbalance by exerting negative feedback on the hypothalamic-pituitary axis [5]. Prolactin elevations, although less common, may impair gonadotropin-releasing hormone secretion, compounding reproductive dysfunction [13]. Molecular biomarkers revealed substantial oxidative and genetic stress in infertile men. The elevated ROS levels observed mirror results from Aitken, who demonstrated that excessive ROS leads to lipid peroxidation of sperm membranes and mitochondrial dysfunction [6]. The concomitant increase in sperm DNA fragmentation in our study supports findings by Agarwal *et al.* who established DNA fragmentation as a predictor of poor fertilization and embryo development [8]. Approximately one-third of our infertile participants had DNA fragmentation exceeding 30%, a threshold linked to recurrent implantation failure. Mitochondrial dysfunction, evidenced by increased membrane potential loss, suggests compromised energy metabolism. Similar findings were reported by Rato *et al.* who emphasized that impaired mitochondrial function undermines sperm motility and fertilization capacity [14]. The upregulation of HSP70 observed in this study likely reflects a stress-response mechanism to protect spermatozoa against oxidative insults [15]. However, persistent overexpression may indicate ongoing cellular distress, potentially contributing to impaired spermatogenesis. Lifestyle and metabolic conditions such as obesity and diabetes appear to aggravate infertility in our population. Infertile men in this study had significantly higher BMI, consistent with Barbagallo *et al.* who linked obesity to increased estrogen levels and oxidative stress [9]. Davidson *et al.* also highlighted the detrimental role of adiposity in hormonal imbalance and sperm dysfunction [16]. In Bangladesh, high prevalence of diabetes and metabolic syndrome likely compounds these issues [11].

The clinical implications of these findings are substantial. Hormonal assays and molecular biomarkers can provide complementary insights into infertility diagnosis. Elevated FSH and LH levels may indicate irreversible testicular failure, guiding clinicians toward assisted reproductive technologies (ART). Conversely, identifying oxidative stress and DNA fragmentation suggests potential benefits from antioxidant therapy and lifestyle modification [17]. The findings of this study align with Bracke *et al.* who emphasized the multifactorial nature of idiopathic male infertility, involving both genetic and molecular disturbances [12]. Genetic predispositions, including Y-chromosome microdeletions, may interact with environmental factors to amplify risk. Emerging research on epigenetic regulation further suggests that altered DNA methylation patterns in sperm can influence embryonic development [18]. By combining hormonal and molecular analyses, our research bridges this gap and highlights actionable diagnostic pathways. Nonetheless, challenges remain. Antioxidant therapies show mixed outcomes in clinical trials, with some studies reporting improvements in sperm DNA integrity and others showing no significant benefit [19]. Similarly, hormonal interventions such as aromatase inhibitors or selective estrogen receptor modulators may help restore testosterone- estradiol balance in selected patients, but long-term efficacy is uncertain [4]. In summary, this study reinforces that male infertility arises from a confluence of hormonal dysregulation, oxidative stress and molecular abnormalities. Clinical strategies should move toward integrated approaches that combine hormonal evaluation, oxidative stress testing and lifestyle interventions. Multicenter longitudinal studies incorporating genomics and proteomics are needed to fully elucidate mechanisms and personalize treatments.

## Conclusion:

We show the male infertility in Bangladesh is associated with significant hormonal imbalances and molecular abnormalities, including elevated gonadotropins, reduced testosterone, oxidative stress and increased DNA fragmentation. Thus, we show the multifactorial nature of infertility, where endocrine dysregulation interacts with cellular and molecular damage. Recognizing these biomarkers may help clinicians refine diagnostic protocols and improve individualized treatment strategies for infertile men.

## Figures and Tables

**Table 1:** Baseline characteristics of study participants (N = 300)

**Variables**	**Infertile Men (n=200)**	**Fertile Controls (n=100)**	**p-value**
Age (years, mean±SD)	32.4±5.6	31.9±5.2	0.47
BMI (kg/m^2^, mean±SD)	27.1±3.4	24.6±2.9	<0.01
Duration of Infertility (years)	3.2±1.8	-	-
Smoking (%)	42 (21%)	14 (14%)	0.09
Diabetes Mellitus (%)	28 (14%)	6 (6%)	0.04

**Table 2:** Hormonal profiles in infertile versus fertile men

**Hormone (Mean±SD)**	**Infertile (n=200)**	**Fertile (n=100)**	**Reference Range**	**p-value**
FSH (mIU/mL)	9.1±3.7	5.6±2.1	1.5-12	<0.001
LH (mIU/mL)	6.4±2.3	4.8±1.9	1.7-8.6	<0.01
Testosterone (ng/mL)	3.1±1.2	5.2±1.6	3-10	<0.001
Prolactin (ng/mL)	17.8±6.5	13.1±4.7	4-20	<0.01
Estradiol (pg/mL)	42.3±14.8	29.5±10.6	10-40	<0.01

**Table 3:** Molecular biomarkers associated with male infertility

**Biomarker**	**Infertile (n=200)**	**Fertile (n=100)**	**p-value**
Sperm DNA Fragmentation Index (%)	32.5±9.1	15.6±6.4	<0.001
ROS Levels (RLU/10^6^ sperm)	6.1±2.3	3.2±1.4	<0.001
Mitochondrial Membrane Potential Loss (%)	41.2±11.7	22.8±8.9	<0.001
HSP70 Expression (Relative Fold Change)	2.7±0.8	1.3±0.5	<0.001

**Table 4:** Hormonal and molecular dysregulation

**Parameter**	**Normal Range**	**Infertile Men with Abnormal Values, n (%)**
Low Testosterone (<3 ng/mL)	3-10 ng/mL	76 (38.0)
Elevated FSH (>10 mIU/mL)	1.5-12 mIU/mL	58 (29.0)
High ROS (>5 RLU/10^6^ sperm)	<5 RLU/10^6^ sperm	88 (44.0)
Elevated Estradiol (>40 pg/mL)	10-40 pg/mL	54 (27.0)
High DNA Fragmentation (>30%)	<25%	72 (36.0)
